# Memory specificity training can improve working and prospective memory in amnestic mild cognitive impairment

**DOI:** 10.1590/1980-57642016dn11-030007

**Published:** 2017

**Authors:** Golita Emsaki, Hamid Taher NeshatDoost, Mahgol Tavakoli, Majid Barekatain

**Affiliations:** 1 PhD Student of Psychology, Department of Psychology, University of Isfahan, Iran.; 2 Professor of Clinical Psychology, Department of Psychology, University of Isfahan, Iran.; 3 Assistant Professor of Psychology, Department of Psychology, University of Isfahan, Iran.; 4 Professor of Neuropsychiatry, Department of Psychiatry, School of Medicine, Isfahan University of Medical Sciences, Iran.

**Keywords:** mild cognitive impairment, memory specificity training, memory, depression, comprometimento cognitive leve, especificidade de treinamento de memoria, memoria, depressão

## Abstract

Amnestic Mild Cognitive Impairment (MCI) is one of the cognitive profiles of aging.

**OBJECTIVE:**

In this study, Memory Specificity Training (MEST) was used as cognitive training in patients with amnestic MCI to understand the effectiveness of the intervention on memory dimensions.

**METHODS:**

Twenty patients that met the criteria for amnestic MCI were selected and randomly assigned to experimental (n=10) or control (n=10) groups. The experimental group received five sessions of training on memory specificity while the participants in the control group took part in two general placebo sessions. Participants were assessed before, immediately after, and three months after, the treatment using the Autobiographical Memory Test, the Prospective and Retrospective Memory Questionnaire, the Wechsler Memory Scale, and the Hospital Anxiety and Depression Scale. Analysis of variance was used to analyze the data.

**RESULTS:**

Results from both post-test and follow-up treatment indicated that MEST improves working and prospective memory (p<0.05).

**CONCLUSION:**

These findings support the effectiveness of MEST for MCI patients as a viable cognitive intervention. Also, the findings have implications for the role of brain plasticity in the effectiveness of this intervention.

## RESUMO

Comprometimento cognitivo leve amnéstico (CCLA) é um dos perfis cognitivos do envelhecimento.

**OBJETIVO:**

*Memory Specificity Training* (MEST) foi usado como treino cognitivo em pacientes com CCLA para compreensão da efetividade da intervenção em dimensões da memória.

**MÉTODOS:**

Vinte pacientes que preencheram critérios para CCLA foram selecionados e randomizados para os grupos experimental (n=10) ou controle (n=10). O grupo experimental recebeu cinco sessões de treinamento em especificidade de memória enquanto que os pacientes do grupo controle participaram em duas sessões placebo. Os participantes foram avaliados antes e imediatamente após o tratamento e, três meses depois, usando o teste de memória autobiográfica, questionário de memória prospectiva e retrospectiva, escala de memória de Weschler e a escala hospitalar de depressão e ansiedade. A análise de variância foi usada para analisar os dados.

**RESULTADOS:**

Ambos os resultados, no pós-teste e no acompanhamento indicaram que o MEST melhora memória prospectiva e de trabalho (p<0.05).

**CONCLUSÃO:**

Estes achados suportam a efetividade do MEST para pacientes com CCL, como uma intervenção viável. Também, os achados têm implicações para o papel da plasticidade na efetividade desta intervenção.

## INTRODUCTION

The diagnosis of mild cognitive impairment (MCI) requires above-average, persistent cognitive impairment without symptoms of dementia. The National Institute on Aging-Alzheimer's Association defines MCI as a slight, yet measurable, cognitive disorder.^1^ MCI is divided into two subcategories: amnestic and non-amnestic domains. Both types include either a single domain or multiple domains. Amnestic single domain MCI only involves memory impairment, whereas non-amnestic MCI patients have complaints involving other cognitive domains. Various manifestations of cognitive impairments can represent different neurodegenerative pathologies. For instance, while amnestic MCI is likely to indicate an early stage of Alzheimer's disease (AD), the non-amnestic domain type may indicate a vascular pathology or frontotemporal dementia.^2^

Amnestic MCI is typically described using the criteria suggested by Petersen (1999), which include memory complaints, objective memory impairment, absence of functional problems in daily life, and absence of dementia.^3^ Petersen et al. suggested that average memory performance on neuropsychological tests for patients with MCI is 1.5 standard deviations below healthy individuals' performance with similar age and education.^3^^-^ 5

The increasing rate of conversion from amnestic MCI to AD has prompted the use of numerous medications, such as acetylcholinesterase inhibitors, to delay or prevent conversion. However, meta-analyses report no delays in progression to dementia in treated patients compared with placebo.^4^^,^ 6 ^,^ 7 Thus, in recent years, non-medicational interventions have received greater attention. More recently, research has shown that MCI patients show adequate levels of neuroplasticity, and are capable of cognitive learning. For example, using fMRI, Belleville et al. (2011) showed that cognitive training can lead to measurable neural changes in the brain of patients with MCI.^8^ These data, and other similar results, support brain plasticity in the elderly and suggest that cognitive interventions might be effective for promoting cognitive improvement in MCI patients.^6^

Cognitive interventions are divided into three categories: cognitive stimulation, cognitive training, and cognitive rehabilitation. Cognitive stimulation involves engagement in group activities designed to increase cognitive and social performance using non-specific approaches. In contrast, cognitive training is a more specific approach which teaches strategies and skills to enhance certain aspects of cognitive performance.^4^^,^ 7 ^,^ 9

Numerous cognitive training techniques have been introduced, most of which are adjusted according to the cognitive status of the patient. As mentioned above, the primary issue in patients with amnestic MCI concerns memory.^3^ Therefore, training which focuses highly on memory could be beneficial for MCI patients.

A commonly affected area of memory in both MCI and AD patients is autobiographical memory. Autobiographical memory is a kind of declarative memory which consists of episodes of an individual's life.^10^ Given the critical role of this type of memory in one's sense of self (i.e. identity), deficiencies are likely to have devastating consequences for patients and their families.^11^

Memory Specificity Training (MEST)^12^ is a technique which aims to reduce over-generalized memories by increasing more specific memories. Preliminary studies suggest that MEST is effective in improving mood in depressed patients.^13^^,^ 14 However, until now, MEST has not been used to improve cognitive domains. There are a number of reasons justifying the application of this technique to improve cognitive performance. First, findings indicate that older individuals tend to remember more general memories compared to their younger counterparts.^15^ Second, the hippocampus is where memory specificity occurs^16^ and it has been shown that disparities in memory performance among older adults may be due to differences in the amygdala-hippocampus circuit.^17^ Also, the medial temporal cortex, and the hippocampus in particular, are the first areas affected in MCI. Therefore, owing to brain plasticity, training patients to remember specific memories could improve their memory because the hippocampus becomes involved.

## METHODS

### Participants.

Twenty patients diagnosed with amnestic MCI who met the criteria were enrolled in the study. All participants were Iranian retired patients who were diagnosed with amnestic MCI according to Peterson's criteria for mild cognitive impairment.^3^ Inclusion criteria for the study were having a diagnosis of amnestic MCI, an absence of intellectual disability, and holding at least a high-school diploma. Furthermore, patients reported no complaints of an inability to perform typical or routine tasks and had no major medical, psychological, or neurological disorders. Patients who were diagnosed with depression or anxiety by a psychiatrist were excluded from the study. Individuals with a history of taking drugs which may cause serious cognitive disorders and inability to function, psychotropic medications or drugs which may hinder cognitive abilities were also excluded. Finally, physical health (i.e. lack of visual and hearing problems) and absence of a dementia diagnosis were required. Following the pre-test, the patients were randomly divided into two groups: MEST and control. The purpose of the latter group was to control for non-specific treatment factors as well as the placebo effect. Average age for the MEST and control groups was 63.1 (SD=5.56) and 63.7 (SD=7.14) years, respectively. Mean education for the MEST group was 14.2 (SD=1.75) years and for the control groups was 14.0 (SD=1.63) years.

### Measures

*The Autobiographical Memory Test (AMT):* Developed in 1986 by Williams and Broadbent. This test includes 18 cue words with positive, negative, or neutral valence. Subjects are presented with a cue word and asked to provide the first memory they remember, which may be related to a significant, trivial, recent, or old event; however, the event must be specific, i.e. associated with a particular time and place and last no longer than one day. Three practice attempts then ensure that subjects are sufficiently familiar with the procedure. Subsequently, the 15 remaining words are presented in a counterbalanced manner with 30 seconds to recall each memory. Subjects are encouraged to provide specific memories. The test has high internal consistency and reliability – an α coefficient of 0.83 as measured by the original authors. A Cronbach's α coefficient of 0.86 has also been reported.^10^^,^ 14 ^,^ 18

*The Prospective and Retrospective Memory Questionnaire (PRMQ):* A self-report questionnaire developed in 2000 by Smith, DellaSala, Logie, and Maylor. This 16-item instrument assesses retrospective and prospective memory slips in everyday life. Each item pertains to one failure which is rated on a five-point scale by the subject. Higher scores indicate more intense failures. As reported by Crawford et al. (2003), both subscales have high reliability: α=0.84 for prospective memory and α=0.80 for retrospective memory.^19^ The PRMQ was also standardized in Iran by Zare, Alipur, & Mostafaie in 2014 and the results showed a high reliability (Cronbach α=0.83) among Iranian population.^20^

*The Wechsler Memory Scale (WMS):* The original version of the WMS was designed by David Wechsler in 1945 to measure memory functions in individuals aged between 19 and 90. The most recent version (i.e. the WMS-IV) was developed in 2009.^21^ In the paper, the Spatial span, Logical Memory, and Verbal Paired Associates (I and II) subscales of the WMS-III are used to assess working and auditory memories while visual memory is examined using the Visual Reproduction and Designs (I and II) subscales of the WMS-IV. The reliability coefficients for most WMS-III subscales fall between 0.82 and 0.93.^21^ In Iran, Saed, Rushan and Moradi (2008) investigated the psychometric properties of the WMS-III and their results showed satisfactory reliability for this scale (Cronbach α for immediate auditory memory index, delayed auditory memory index and working memory were 0.83, 0.8 and 0.8, respectively).^22^ The authors of the WMS-IV report an α coefficient of 0.95 to 0.97 and internal consistency of 0.8 for the visual memory index.^23^

*The Hospital Anxiety and Depression Scale (HADS):* proposed by Zigmond and Snaith in 1983, the HADS was designed to detect potential anxiety and depression in non-psychiatric patients. It consists of two seven-item subscales: anxiety and depression. Since physical disorders are not considered in scoring, the items fail to include physical symptoms of anxiety and depression such as vertigo, headaches, sleep problems, fatigue, and exhaustion.^24^ In this study, only the depression subscale was administered. In a review of 747 papers on the HADS, Bjelland, Dahl, Haug, & Neckelmann, (2002) reported internal consistency and sensitivity of the depression subscale of 0.83 and 0.8, respectively. Furthermore, the correlation of the subscale with other scales of depression ranged from 49 to 83 percent.^25^ The validity and reliability of the HADS have been investigated among Iranians by Kaviani, Seyfourian, Sharifi, & Ebrahimkhani in 2009, and the results showed high reliability and validity for the measure and its subscales (r=0.77 with the BDI, r=0.81 with the BAI, Cronbach α=0.7 for the depression subscale and 0.85 for the anxiety subscale).^26^

### Design and procedure.

**Figure 1:**
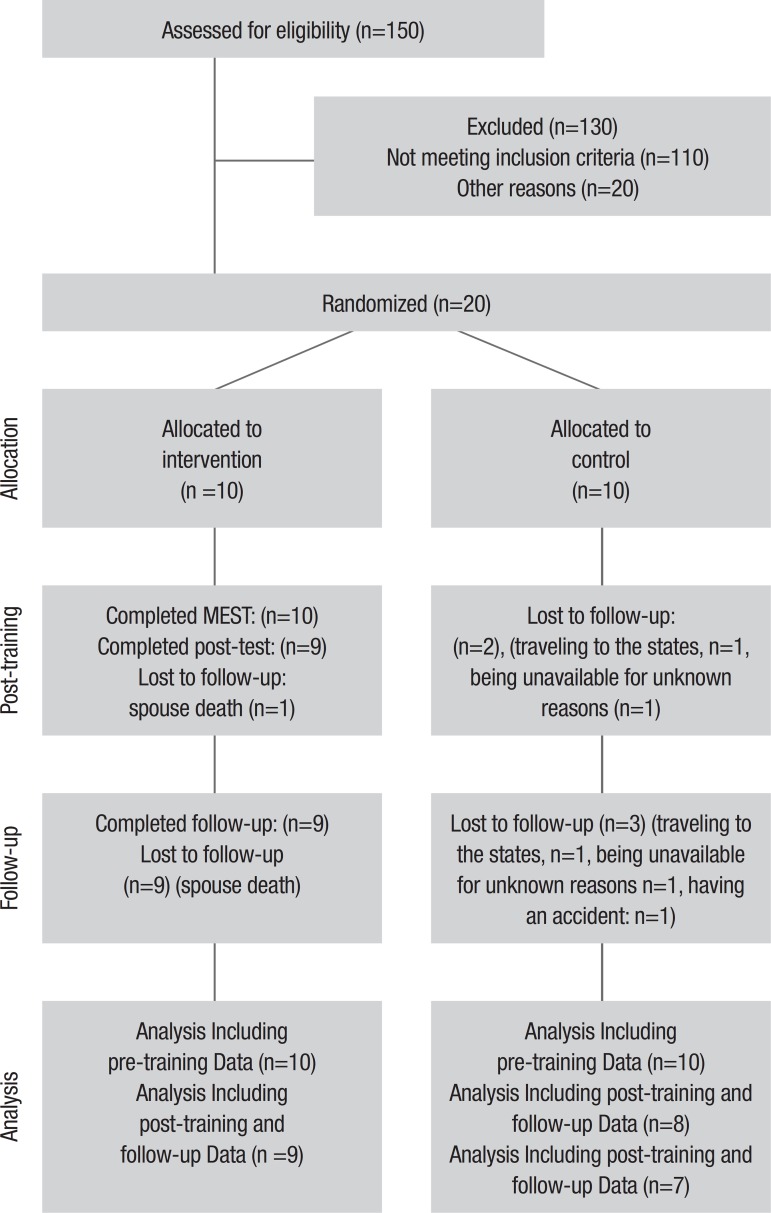
CONSORT (consolidated standards of reporting trials) diagram of progress through phases of the randomized trial.

Participants were tested individually by one of the researchers at pre-training, post-training, and 3 months after training. It should be noted that the Isfahan University of Medical Sciences Code of Ethics was followed in conducting the study and all patients signed informed consent forms. Figure 1 depicts the CONSORT (consolidated standards of reporting trials) diagram for the trial.

Five weekly 80-minute sessions of MEST were administered. The first session provided a definition of amnestic MCI and its distinction from natural aging. Brain plasticity, memory problems and the frameworks of treatment were also discussed. Additionally, patients were introduced to extended, specific, and categorical recall and were asked to produce specific memories in response to a given set of words that covered all three types. The next session concerned positive words and the associated memories; the same process was performed for negative and neutral words in the subsequent sessions. Finally, in the fifth session, the materials were reviewed and the difference between autobiographical memory recall with positive, negative, and neutral words was discussed.^14^ Patients in the control group participated in two sessions educating about amnestic MCI symptoms, how it differs from natural aging, and patients' memory problems. All patients in the MEST group completed a post-test with a three-month follow-up, after which subjects in the control group were given all the pertinent educational material about memory specificity training. Ethical approval was obtained from the University of Isfahan.

## RESULTS

Mean and standard deviation for age and education of all the participants is reported. In order to compare the mean of memory and depression scores of the two groups in post-test and follow-up, between-group comparisons were carried out using multivariate ANOVA. All data were analyzed using the SPSS (version 21) statistics software package. The significance level for all of the hypotheses was set as 0.05.

After controlling for pre-test scores, Multivariate Analysis of Variance (MANOVA) was applied to assess average patient scores in both groups at post-test and follow-up. Prior to the analyses, assumptions of normality (Shapiro-Wilk test) and homogeneity of variances (Levene test of homogeneity of variance) were assessed and confirmed for all the variables, allowing the application of parametric MANOVA.

Different dimensions of memory and two demographic variables (i.e. age and education) were found to have no significant correlations (using Pearson correlation coefficient, p<0.05), thus eliminating the need to control for the variables in MANOVA.

Table 1 shows Mean and SD of memory and depression scores at pre-test, post-test and follow-up and Table 2 shows the results of MANOVA at post-test and follow-up stages for each of the groups.

**Table 1:** Mean and standard deviation for memory and depression scores at pre-test, post-test and follow-up.

	Group	Mean (SD)				n		
		Pre-test	Post-test	Follow-up		Pre-test	Post-test	Follow-up
Visual immediate	MEST	60.6 (9.74)	61.22 (11.92)	62.22 (10.06)		10	9	9
	Control	63.4 (14.29)	67.16 (13.79)	65.33 (14.1)		10	8	7
Visual delayed	MEST	29.8 (15.92)	29.77 (16.92)	29.44 (16.46)		10	9	9
	Control	29.5 (16.53)	31.83 (17.44)	32.16 (16.96)		10	8	7
Auditory immediate	MEST	21.6 (8.46)	22.55 (9.22)	22.44 (8.93)		10	9	9
	Control	17.7 (3.23)	18 (5.62)	17.66 (4.22)		10	8	7
Auditory delayed	MEST	12.2 (5.73)	12.66 (5.48)	12.44 (5.59)		10	9	9
	Control	11.5 (2.36)	12.89 (3.87)	13 (2.97)		10	8	7
Working	MEST	11.3 (2.98)	14 (3.94)	14.33 (4.55)		10	9	9
	Control	12.2 (3.08)	11.66 (3.72)	11 (3.03)		10	8	7
Retrospective	MEST	50.4 (2.87)	50.11 (3.62)	50.55 (3.04)		10	9	9
	Control	44.4 (3.77)	44 (6.32)	41.83 (6.94)		10	8	7
Prospective	MEST	49.4 (2.84)	51.33 (4.12)	51.22 (4.76)		10	9	9
	Control	47 (4.42)	45.66 (4.84)	44.5 (5.68)		10	8	7
Autobiographical	MEST	9.8 (1.85)	12.77 (1.92)	11.88 (1.96)		10	9	9
	Control	8.7 (2.21)	8.57 (1.9)	8.58 (1.51)		10	8	7
Depression	MEST	5.6 (3.27)	4.33 (2.5)	4.55 (2.96)		10	9	9
	Control	5.5 (2.71)	5 (3.9)	5.33 (2.34)		10	8	7

**Table 2:** ANOVA for investigating difference in memory and depression scores between MEST and Control groups at post-test and follow-up.

Source	Dependent variables	df	Mean square	F	p	Partial eta square	Observed power
Group	Immediate visual post-test	1	1.732	0.24	.628	0.02	0.74
	immediate visual follow-up	1	22.29	2.51	0.14	0.17	0.309
	Delayed visual post-test	1	4.278	0. 380	0.549	0. .031	0. 088
	Delayed visual follow-up	1	0. .399	0. .033	0. 859	0. 003	0. 053
	Immediate auditory post-test	1	6.900	0. 504	0. 491	0. 040	0. 100
	Immediate auditory follow-up	1	10.978	1.134	0. 308	0. 086	0. 166
	Delayed auditory post-test	1	0. 398	0.100	0. 757	0. 008	0. 060
	Delayed auditory follow-up	1	2.984	1.307	0. 275	0. 098	0. 184
	Working post-test	1	37.557	12.809	0. 004	0. 516	0. 907
	Working follow-up	1	63.725	13.122	0. 003	0. 522	0. 913
	Retrospective post-test	1	0. 123	0. 011	0. 917	0. 001	0. 051
	Retrospective follow-up	1	10.950	0. 925	0. 355	0. 072	0. 144
	Prospective post-test	1	29.043	5.661	0. 035	0. 321	0. 590
	Prospective follow-up	1	42.737	5.900	0. 032	0. 330	0. 607
	Autobiographical post-test	1	65.135	24.263	0. 000	0. 651	0. 995
	Autobiographical follow-up	1	40.142	16.683	0.001	0. 562	0. 965
	Depression post-test	1	2.531	0. 766	0. 399	0. 060	0. 127
	Depression follow-up	1	3.248	3.587	0. 083	0. 230	0. 414

* p<.05.

The results of MANOVA showed that, even after controlling for the effect of pretest, there was a significant difference between the two groups in working memory (F=12.809, p=0.004 at post-test and F=13.122, p=0.003 at follow-up), prospective memory (F=5.661, p=0.035 at post-test and F=5.9, p=0.032 at follow-up) and in autobiographical memory (F=24.26, p=0.00 at post-test and F=16.68, p=0.001 at follow-up) at both post-test and follow-up. The pertinent observed power showed that the sample size sufficed for these conclusions.

## DISCUSSION

This paper aimed to investigate the impact of MEST on memory and mood in patients with amnestic MCI. The results revealed that MEST influences working and prospective memories while increasing the number of specific autobiographical memory recalls in the experimental group. The results remained unchanged during the three-month follow-up period. In contrast, no significant effects were found with respect to other dimensions of memory.

In cognitive training, individuals are given a set of assignments whose goal is to improve underlying impaired cognitive functions.^27^ The theory of complementary learning systems asserts that the hippocampus is responsible for distinct reproductions of one's memories (i.e. specific memories). However the cortex abstracts the shared aspects of these memories.^28^ Therefore, a reduction in specific memories is expected in amnestic MCI as the medial temporal lobe begins to degenerate and atrophy.^29^ It has been shown that there is an interaction between the amygdala and medial temporal lobe, especially the hippocampus, during triggering of emotional memories. FMRI studies demonstrated higher activity in the amygdala during recollection of emotional memories which led to enhanced activity in the medial temporal lobe.^30^^,^ 31

A recent study (e.g. Young et al., 2015) showed that MEST results in hyperactivity of the amygdala and hence stimulates change in the hippocampus and medial temporal lobe;^32^ this can improve working and prospective memories which are impaired as a result of damaged middle temporal cortex.

This change can be explained by the notion of brain plasticity: the structures and functions of neurons and circuits in the brain change in response to experiences; the ability is even observed in older adults. According to the hippocampal generation phenomenon, hippocampal neurogenesis in adults occurs in response to a variety of sensory, motor, and cognitive stimuli. It may be considered a form of activity-dependent brain plasticity wherein both new synoptic connections and neurons are created.^33^ This fact can explain hippocampus-related improvements of memory in response to cognitive stimulation.

Although we failed to find statistical significant differences in mood scores at pre-test, post-test and follow-up detected in other studies investigating the efficacy of this treatment among depression patients,^14^^,^ 34 a trend in scores of patients between post-test and follow-up was detected. A partial eta squared of 0.23 for depression at follow-up and the significance level of 0.08 demonstrate that, with a larger sample size, the results could be different.

Also, no significant differences were observed in visual and auditory memories, whether immediate or delayed. It seems that interventions which directly manipulate the visual and auditory dimensions of memory and contain exercises directly targeting these dimensions (e.g. cognitive rehabilitation methods) are more likely to enhance these dimensions.

However, as mentioned earlier, the eligibility criteria to enter the study included having at least high-school education and absence of comorbid diseases; however, many older adults lack proper education and often have other health problems. Therefore, caution should be exercised in generalizing the results to these individuals. As another limitation, lack of access to brain imaging techniques precluded structural and functional comparison of brain activities in MEST and control conditions before and after the intervention.

Overall, MEST can be categorized as a cognitive training method. The treatment is short-term and patients tend to relate well as they are engaged with their personal memories. However, further research is required to establish its efficacy for different groups of patients and diseases. Our findings can also help future studies develop novel interventions for elderly care.
